# Vocal-Tract Resonances as Indexical Cues in Rhesus Monkeys

**DOI:** 10.1016/j.cub.2007.01.029

**Published:** 2007-03-06

**Authors:** Asif A. Ghazanfar, Hjalmar K. Turesson, Joost X. Maier, Ralph van Dinther, Roy D. Patterson, Nikos K. Logothetis

**Affiliations:** 1 Max Planck Institute for Biological Cybernetics, 72076 Tuebingen, Germany; 2 Centre for the Neural Basis of Hearing, Department of Physiology, University of Cambridge, CB2 3EG Cambridge, United Kingdom

**Keywords:** SYSNEURO

## Abstract

Vocal-tract resonances (or *formants*) are acoustic signatures in the voice and are related to the shape and length of the vocal tract. Formants play an important role in human communication, helping us not only to distinguish several different speech sounds [1], but also to extract important information related to the physical characteristics of the speaker, so-called *indexical cues*. How did formants come to play such an important role in human vocal communication? One hypothesis suggests that the ancestral role of formant perception—a role that might be present in extant nonhuman primates—was to provide indexical cues [2–5]. Although formants are present in the acoustic structure of vowel-like calls of monkeys [3–8] and implicated in the discrimination of call types [8–10], it is not known whether they use this feature to extract indexical cues. Here, we investigate whether rhesus monkeys can use the formant structure in their “coo” calls to assess the age-related body size of conspecifics. Using a preferential-looking paradigm [11, 12] and synthetic coo calls in which formant structure simulated an adult/large- or juvenile/small-sounding individual, we demonstrate that untrained monkeys attend to formant cues and link large-sounding coos to large faces and small-sounding coos to small faces—in essence, they can, like humans [13], use formants as indicators of age-related body size.

## Results

Though the whole acoustic spectrum of vowel sounds is ideal for our categorization of speech [14], the lowest-dimensional representations rely on vocal-tract resonances, or *formants* [15]. Formants are not only important phonetic elements of speech—allowing us to distinguish different vowel sounds—but also carry important information related to the physical characteristics of the particular speaker. In humans, both statistical pattern recognition [16, 17] and psychophysics [13, 18–23] have suggested that formants are significant contributors to these indexical cues. It is likely, then, that detecting formants could have provided ancestral primates with indexical cues necessary for navigating the complex social interactions that are the essence of primate societies. One important indexical cue is body size. Formant cues related to body size could be used by monkeys to determine the sex (in sexually dimorphic species), degree of potential threat (e.g., whether a competitor is larger or smaller), and/or age of an individual, as such cues do for human listeners [13, 18, 20, 21].

Formants are the result of acoustic filtering by the supralaryngeal vocal tract—the nasal and oral cavities above the vocal folds. During vocal production, pulses of air generated by the rapid movement of the vocal folds produce an acoustic signal. The frequency of these pulses—the glottal-pulse rate—determines the fundamental frequency of the signal, which in turn is perceived as pitch. As the signal passes through the supralaryngeal vocal tract, it excites resonances, resulting in the enhancement of particular frequency bands; these are the formants. The length of the vocal tract determines, in part, which frequency bands are enhanced [2, 15]: The frequency of, and the spacing between, successive formants decreases with *increasing* vocal-tract length. Because the vocal-tract length scales with body size in humans [24], formants are often reliable cues to this physical feature [13, 18, 20, 21].

**Figure 1:**
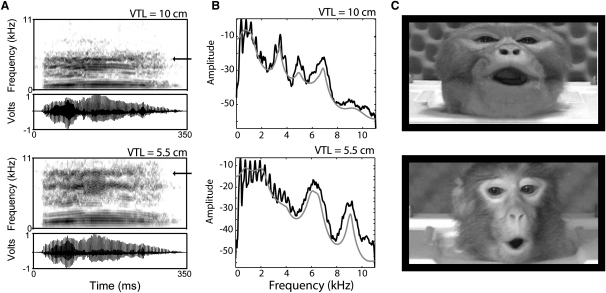
Auditory and Visual Stimuli Used in the Current Experiments (A) Resynthesized coo vocalizations based on one of the two coo exemplars used in the preferential-looking paradigm. Diagram shows the spectrograms and waveforms of a coo vocalization resynthesized with two different vocal-tract lengths. The arrow in the spectrogram indicates the position of an individual formant, which increases in frequency as the apparent vocal-tract length decreases. (B) Power spectra (black line) and linear predictive coding spectra (gray lines) for the long vocal-tract length (10 cm, top panel) and short vocal-tract length (5.5 cm, bottom panel) used in the experiment and seen in (A). (C) Still frames extracted from the videos used in the preferential-looking experiments. The top row shows frames from the large monkey. Videos were synchronized and edited so that they appeared to be synchronously producing the coo vocalization shown in (A).

Acoustic analyses of rhesus monkey vocalizations reveal that these calls also have prominent formant structure [3, 5, 25] and that this spectral structure could, in theory, provide monkeys with indexical cues about their conspecifics, including information about their body size [3]. Here, we explicitly test the hypothesis that rhesus monkeys use formants as salient acoustic cues to assess the age-related body-size differences of conspecifics. A direct, experimental approach for assessing the role of formants includes the use of vocal synthesis methods in which the formant frequencies of a call can be manipulated independently of other acoustic cues (e.g., the fundamental frequency [glottal-pulse rate]) [26]. Only recently have such synthetic vocalizations been used successfully in animal playback experiments (whooping cranes [27], red deer [28], and rhesus monkeys [29]). Along similar lines, we used naturally produced rhesus monkey “coo” calls as models and a speech vocoder [30] to synthesize versions of these calls in which the glottal-pulse rate and all other acoustic variables (e.g., duration and amplitude envelope) were held constant while the formant frequencies were shifted up or down. Figure 1A shows the spectrograms of a single coo synthesized with two different vocal-tract lengths (10 cm and 5.5 cm). Note how the formants shift down and become more concentrated for large vocal-tract lengths and shift up and spread out for short vocal-tract lengths, whereas the overall shape of the amplitude envelope remains unchanged. The shift in formant spacing is also evident in the power and linear prediction spectra for the two vocal-tract lengths (Figure 1B).

To determine whether rhesus monkeys use formant cues to assess age-related differences in conspecific body sizes, we adopted a preferential-looking paradigm. Previous work has established that, like human infants (e.g., [31, 32]), rhesus monkeys naturally prefer to look at a visual stimulus that corresponds to the auditory stimulus that they hear [11, 12]. In the present context, we tested whether our monkey subjects would preferentially attend to a video display showing a large, older monkey (sexually mature, 13-yr-old) versus a small, younger monkey (juvenile, 6-yr-old) producing a coo vocalization (Figure 1C) when they heard a coo produced from a simulated long vocal tract, and vice versa (Figures 1A and 1B). Monkeys were seated in front of two LCD monitors and a hidden speaker located between them and at the same height. One monitor displayed a video of the face of the large monkey producing a coo call, and the other monitor displayed the face of the small monkey producing a coo call. We counter-balanced all pertinent variables in the experiment. Both videos were played synchronously in a continuous loop for 60 s. Videos were edited such that the onset and offset of each monkey's mouth movements was synchronous. Synchronously with the videos, the subjects heard a coo that was from a long vocal tract (10 cm) or a short vocal tract (5.5 cm) and was based on a call from a third individual (Figures 1A and 1B). The call of this third individual was based on one of two coo calls from other individuals of different ages (a sexually mature, 11-yr-old adult and a juvenile 6-yr-old) to eliminate any chance that the subjects could match the call with the dynamic faces by using some other individual-specific articulatory cue or some age-related acoustic cue(s) independent of formants.

Although only the heads were visible in these videos, subjects could putatively assess overall size by features of the face (their size or the relative positions of facial features) or by comparing the head size relative to parts of the chair in which the vocalizing monkeys were seated (Figure 1C). Head size can be used as a proxy for overall body size because there is a strong correlation between skull size and body size (as measured by either weight or length) and thus with vocal-tract length and formant spacing [3]. Because all visual and auditory components were synchronized and identical in both duration and overall amplitude, amodal cues could not be used to make a match. Two sets of such audiovisual stimuli were generated and used in these experiments; that is, there were two coo calls that were from two differently aged and sized individuals and were manipulated to sound large and small and then paired to the videos. Thus, our paradigm addressed whether monkeys would preferentially attend to the dynamic face that was approximately matched in size to the coo call that simulated that body size.

**Figure 2:**
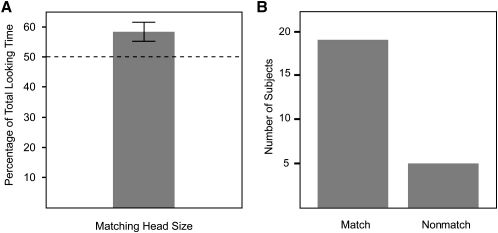
Monkeys Match the Acoustic Size Extracted from Formant Frequencies to the Matching Face (A) The mean percentage of total looking time spent looking at the matching video display; the dotted line indicates chance expectation (n = 24). Error bars represent the standard error of the mean. (B) A significant proportion of subjects looked longer at the match than the nonmatch screen.

Monkeys looked at the matching screen for 58.4% of the total time they spent looking at either screen (match: 13.08 ± 1.45 s; nonmatch: 10.26 ± 1.49 s); this proportion differed significantly from chance [one-sample t test, t(23) = 2.67, p = 0.014] (Figure 2A). This ∼3 s difference, although seemingly small, is robust in the context of the preferential-looking method and is similar to differences reported for similar experiments in both humans [31, 32] and monkeys [11, 12]. With the percentage of total looking time to the match screen used as a dependent variable, an ANOVA was conducted to explore any possible interactions among four primary variables (side of screen [left versus right], vocalizer [acoustic signal of monkey 1 versus monkey 2], face [visual signal of monkey 1 versus monkey 2], and vocal tract length [long versus short]). All main effects or interactions were nonsignificant. Thus, there were no response biases toward the left or right screen, the stimulus exemplars (the calls or the faces used), or the size of the monkey on the matching screen (e.g., monkeys did not look longer overall when the matching screen showed a large monkey). Nineteen out of twenty-four monkeys in the present experiment preferentially attended to the dynamic face that best matched the body size simulated by the coo vocalization played through the speaker (Figure 2B, sign test, p = 0.003). These results demonstrate that rhesus monkeys can, without any training whatsoever, use formant structure to assess the age-related body size of conspecific individuals.

## Discussion

Previous behavioral studies demonstrated that trained baboons [33] and macaques [34–36] can discriminate different human vowel sounds presumably on the basis of formant-frequency differences. Recently, Fitch and Fritz [29] have significantly extended these findings by showing that rhesus monkeys can, without training, discriminate differences in the formant structure of their own conspecific calls. However, a demonstration that particular sorts of features appear in species-typical vocalizations or that animals can attend to such features is (though of great importance) not equivalent to showing them to be functionally significant to the animals in question. The functional significance of formants in monkey vocalizations was first suggested by the study of Owren [9, 10], who showed that trained vervet monkeys could use formants to distinguish between their alarm calls (akin to the way in which humans may discriminate speech sounds). The results of our experiments suggest that rhesus monkeys can not only spontaneously discriminate changes in formant structure within a call type (à la [29]), but can also use these differences in formant structure as indexical cues—to assess the age-related size of a conspecific individual. Although body size is just one indexical cue among many that may be encoded in the formant frequencies of monkeys, our data show that, as in humans [13, 18, 20, 21], acoustic cues that are the product of vocal-tract length can be used to estimate body size. These data are the first direct evidence for the hypothesis that formants embedded in the acoustic structure of nonhuman primate calls provide cues to the physical characteristics of the vocalizer [3–7].

Rhesus monkeys and humans are not alone in this regard. One other nonhuman species perceives a link between formant structure and body size: red deer, *Cervus elaphus*. Recent studies of red deer males during their mating season show that not only do red deer roars contain formant structures that are indicators of a male's body size and fitness [37], but male red deer are also more attentive and, in some cases, will reply with more roars when they hear synthetic male roars with lower formant frequencies (simulating a large stag) [28]. Indeed, red deer are able to “exaggerate” their apparent size by actively lowering their larynx during vocal production, thereby creating a longer vocal tract (and thus lower formant frequencies) [38]. Nonhuman primates are not known to be able to actively lower their larynx in this manner during vocal production. Taken together, the fact that rhesus monkeys and red deer can both use formant cues to assess body size begs the question: Is their common perceptual ability the result of convergent evolution (i.e., they evolved independently) or common ancestry (i.e., all or most mammals share this capacity)?

If all mammals were endowed with the capacity to assess body-size cues (age-related or otherwise) via formant frequencies, then it would suggest that even in mammals whose own vocalizations lack formant structure, formant discrimination would still be evident. For example, in small mammals (including small primates, such as New World marmosets or squirrel monkeys) that have short vocal tracts and high frequency calls, formant structure is simply not present in their vocalizations (see [29] for details regarding why this is so) and thus formant perception in these animals would exist without purpose, perhaps as the nonadaptive by-product of other auditory mechanisms. The alternative evolutionary scenario would suggest that the link between formant perception and indexical cuing arose in parallel, possibly multiple times during the course of mammalian evolution. Indeed, the divergent vocal *production* apparatuses between primates and red deer suggest that the evolution of vocal communication among mammals did not take a linear, unbranching path. Naturally, a direct test of either of these hypotheses would entail exploring formant perception in untrained animals that lack formant structure in their own vocalizations.

Regardless of the evolutionary origins of acoustic body-size perception via formants, the link between rhesus monkey perception and human perception is likely to be direct because they are closely related species. However, in human speech perception, indexical cues are coupled with phonetic cues. Humans are able to identify vowel sounds across a wide range of speaker body sizes and ages (and thus different formant-frequency positions), though it is not a feature we consistently attend to. Nevertheless, recent human psychophysical studies revealed that humans, when asked, can make accurate judgments of a speaker's body size by using the formant structure embedded in speech sounds [13, 21] and can recognize vowel sounds even when the simulated vocal-tract length is extended beyond the species-typical range [21]. Thus, assessing speaker size through formants may be an automatic, unconscious process that the human auditory system does in everyday speech communication. Even more pertinent to the current findings with rhesus monkeys, humans can use formant frequencies to determine the age category of speakers (juvenile versus adult) [13], and when fundamental frequency is put into conflict with formant information, human listeners rely on the formants to make age judgments [13].

A question that remains open is whether monkeys and/or humans *within* the category of adults can use formant cues to assess body size. Theoretically, such an assessment could be useful in male-male competition or mate attraction (as in the red deer, described above). Behavioral and acoustic evidence for either scenario, however, remains somewhat ambiguous. For example, in humans, acoustic measurements reveal a relationship only between adult-male height and formant spacing [17, 22], whereas others find a significant correlation only between female height and formant spacing [23, 39]. At the behavioral level, these cues may not be sufficient for assessing speaker size [22]. The reasons for these apparent inconsistencies across studies are multifarious and possibly include differences in body-size variables measured and speech tokens used, and/or large variation in vocal-tract morphology. Similarly, acoustic measures of formant spacing in the grunt calls of adult-female baboons reveal that it is not reliably correlated with many different measures of body size [6], and no behavioral tests of adult body-size perception via formants in monkeys have been forthcoming. Thus, although formant spacing may be a reliable perceptual cue to body size *across* age classes (as in the present study), this may not be true within an age class.

Given that neither the vocal apparatuses nor brains of human ancestors fossilize, the comparative method is the only way to investigate the evolution of primate communication [40, 41]. By comparing the vocal behavior of extant primates with human communication, one can deduce the behavioral and neural capacities of extinct common ancestors, allowing the identification of homologies and providing clues as to the adaptive functions of such behaviors. The close relationship between Old World macaques and humans allows for putative homologous brain mechanisms related to formant perception to be explored and compared between these species. Our data show that rhesus monkeys can intermodally match the auditory size embedded in their coo calls with the appropriately sized visual image of a vocalizing monkey's face; this ability is independent of the identity of the seen and heard monkey. Thus, monkeys are extracting a size cue from auditory structure alone and subsequently matching it to an appropriately sized visual signal. Could auditory cortex integrate such “high-level” bimodal signals [42], perhaps on the basis of implicit multisensory associations formed during everyday social interactions [43]? A first step would be to demonstrate that particular regions of auditory cortex are sensitive to formant structure relative to other acoustic parameters. A recent human neuroimaging paper revealed that regions adjacent to, but not within, Heschl's gyrus are sensitive to formant differences related to speaker size [44], and we have preliminary neurophysiological data that some cortical sites in the lateral belt (putatively a homologous area) of monkeys are also sensitive to vocal-tract-length-related changes in formant spacing relative to changes in fundamental frequencies (C.F. Chandrasekaran, R.V.D., R.D.P., N.K.L., and A.A.G., unpublished data). It is not known whether neurons in these areas integrate auditory and visual size information; if so, it would be strong evidence that these neurons encode ethologically relevant size information.

It is a long trajectory from body-size perception to speech perception via formant cues. There are many aspects of vocal production that are unique to humans and allow us to produce a broader range of sounds with greater complexity [15]. Our data suggest that the use of formant cues in the perception of vowel sounds by humans in a linguistic context emerged gradually, perhaps for other functional reasons, over the course of human evolution. Perception of indexical cues, such as age-related body size, via formants in vocalizations may be one functional link between the vocalizations of human and nonhuman primates.

## Experimental Procedures

### Subjects

We tested male rhesus macaques (n = 24; age range 4–14 yr) from a large colony housed at the Max Planck Institute for Biological Cybernetics. Animals are socially housed and provided with enrichment objects (toys, hammocks, ropes, etc.). All experimental procedures were in accordance with the local authorities (Regierungspraesidium) and the European Community (EUVD 86/609/EEC) standards for the care and use of laboratory animals. For the purposes of the current experiments, subjects were free-fed food and water.

### Stimuli

The stimuli were digital-video recordings of seated rhesus monkeys spontaneously producing coo vocalizations in a sound attenuated room (Figures 1A and 1B). The stimulus set was based on 3-yr-old digital videos of now-deceased male monkeys from the Max Planck Institute for Biological Cybernetics. These videos were then acquired onto a computer and manipulated as needed in Adobe Premiere 6.0 (www.adobe.com). We extracted the audio track from the digital-video samples. Calls were acquired at 32 kHz and then upsampled to 44.1 kHz to allow playback on our hardware.

To generate synthetic rhesus monkey coo calls, we used computational algorithms previously used in similar studies with human speech sounds [13, 21]. The stimuli used in the present experiments were based on natural rhesus monkey coo calls that had been scaled with STRAIGHT, a speech-processing routine that dissects and analyzes an utterance with glottal-cycle resolution. STRAIGHT produces a pitch-independent spectral envelope that represents the vocal-tract information independent from the source (the glottal pulse or vocal-fold vibrations) [30]. Once STRAIGHT has segregated a coo call into source (the glottal-pulse rate component) and vocal-tract information (the spectral envelope), the coo can then be resynthesized with the spectral envelope contracted or expanded (simulating increases or decreases in vocal-tract length, respectively) or the source information expanded or contracted. The two operations are largely independent. Thus, coo calls produced by a small monkey can be transformed to sound like those of a large monkey, and vice versa, by manipulating the apparent size of the vocal tract while keeping the source constant.

For the experimental paradigms described below, we used two different coo-call exemplars from two differently aged monkeys—a 6-yr-old juvenile monkey, weighing 5.8 kg, and a sexually mature 11-yr-old adult monkey, weighing 10.0 kg. These were our base stimuli. This was done to control for any cues that may be related to body size beyond the resonance frequencies of different vocal-tract lengths. For both calls, we then normalized the glottal-pulse rate to 420 Hz. This was done to control for any acoustic cues to body size that may be related to vocal-fold thickness and glottal-pulse rate. For each of the two vocalizations, we then manipulated its spectral envelope to create two synthetic versions for each call. One version simulated a large monkey with a vocal-tract length of 10 cm, and the other simulated a small monkey with a vocal-tract length of 5.5 cm. These vocal-tract lengths are within the species-typical range for rhesus monkeys [3]. All vocal stimuli were calibrated to the same average root-mean square (RMS) power with Adobe Audition.

### Preferential-Looking Paradigm

Two videos were edited, one of which showed a large monkey (13-yr-old, 9.0 kg) producing a coo vocalization and the other showed a small monkey (6-yr-old, 5.9 kg) producing the same call. One of the synthetic coo vocalizations (see above) simulating either a short or long vocal-tract length was then used to replace the original sound track. The videos were edited in Adobe Premiere such that the onset and offset of mouth movements occurred at exactly the same time. Thus, from spatiotemporal point of view, both monkeys appeared to be producing the same coo call. Two sets of such videos were made for each of the two coo calls used.

The “big monkey” versus “small monkey” visual stimuli were played simultaneously on side-by-side 15 inch LCD monitors (Acer FP559, www.global.acer.com). Audio tracks were synchronized with both videos and played through a hidden speaker (same as above) placed directly between and slightly behind the monitors. The RadLight 3.03 Special Edition software video player (www.radlight.net) was used to play the videos in synchrony. Sounds were presented at an intensity of 72–75 dB (A-weighted) sound-pressure level (SPL) as measured with a Brüel & Kjær 2238 Mediator sound-level meter (www.bksv.com) at a distance of 72 cm. For testing, a subject was brought to the testing room and placed in front of the two monitors at a distance of 72 cm. The monitors were 65 cm apart (center-to-center distance) and at eye level with the subject. All trials were videotaped by a digital-video camera placed above and between the monitors. All equipment was concealed by a thick black curtain except for the monitor screens and the lens of the camera. The experimenter monitored subject activity from outside of the room. During this time, the subject's attention was directed to the center by the flashing of a 1.2W light placed centrally between the two monitors. A test session began when the subject looked centrally. A trial consisted of the two videos and one of the auditory stimuli played in a continuous loop for 60 s. The left-right position of the two videos was counter balanced. Each subject was only tested once, and all trials were recorded on digital video. We used a between-groups design because, as in all studies that examine the spontaneous behavior of animals and prelinguistic human infants, the subjects often quickly habituate to the testing environment. No reward or training was provided.

We collected high-quality, close-up digital videos of the subjects' behavior with a JVC GR-DVL805 digital camera (www.jvc.com). Videos were acquired at 30 frames/s (frame size: 720 × 480 pixels) onto a PC by using an IEEE 1394a input and Adobe Premiere 6.0 software (www.adobe.com). Clips for analysis were edited down to 60 s, starting with the onset of the auditory track. The total duration of a subject's looking toward each video (left or right) was recorded and expressed as the proportion of total time spent looking at either screen. Scoring which of the screens the monkey subjects were looking toward was unambiguous. The screens are far apart in the horizontal dimension, fairly close to the monkey's face, and at eye level. Thus, the monkey has to make large eye and head movements to look to one screen or the other, and it is similarly clear when he is not looking at either screen. To validate this, we had all the videos scored by a second observer blind to the experimental condition in order to determine interobserver reliability, which was 0.938 (p < 0.0001) as measured by a Pearson *r* test. The statistical tests and plotted data are derived from the blind observer's video scores.
